# Homozygous GRHPR C.494G>A mutation is deleterious that causes early onset of nephrolithiasis in West Bengal, India

**DOI:** 10.3389/fmolb.2022.1049620

**Published:** 2022-12-22

**Authors:** Arindam Chatterjee, Kunal Sarkar, Sarbashri Bank, Sudakshina Ghosh, Dilip Kumar Pal, Siddharth Saraf, Dhansagar Wakle, Bidyut Roy, Santanu Chakraborty, Biswabandhu Bankura, Debprasad Chattopadhyay, Madhusudan Das

**Affiliations:** ^1^ Department of Zoology, University of Calcutta, Kolkata, India; ^2^ Department of Zoology, Vidyasagar College for Women, Kolkata, India; ^3^ Department of Urology, Institute of Post Graduate Medical Education and Research, Kolkata, India; ^4^ Human Genetics Unit, Indian Statistical Institute, Kolkata, India; ^5^ Medical College, Kolkata, India; ^6^ ICMR Virus Unit, ID & BG Hospital, Kolkata, India; ^7^ ICMR-National Institute of Traditional Medicine, Belgavi, India; ^8^ School of Health Sciences, NSHM Knowledge Campus, Kolkata, India

**Keywords:** kidney stone disease (KSD), whole exome sequencing (WES), nephrolithiasis (NL), GRHPR, hyperoxaluria

## Abstract

Pediatric nephrolithiasis (NL) or Kidney stone disease (KSD) is an untethered topic in Asian population. In Western countries, the annual incidence of paediatric NL is around 6–10%. Here, we present data from West Bengal, India, on lower age (LA, 0–20 years) NL and its prevalence for the first time. To discover the mutations associated with KSD, twenty-four (18 + 6) rare LA-NL patients were selected for Whole Exome Sequencing (WES) and Sanger sequencing, respectively. It was found that GRHPR c. 494G>A mutation (MZ826703) is predominant in our study cohort. This specific homozygous mutation is functionally studied for the first time directly from human peripheral mononuclear cell (PBMC) samples. Using expression study with biochemical activity and computational analysis we assumed that the mutation is pathogenic with loss of function. Moreover, three genes, AGXT, HOGA1 and GRHPR with Novel variants known to cause hyperoxaluria were found frequently in the study cohort. Our study analyses the genes and variations that cause LA-NL, as well as the molecular function of the GRHPR mutation, which may serve as a clinical marker in the population of West Bengal, Eastern India.

## 1 Introduction

Nephrolithiasis (NL) or kidney stone disease (KSD) is a growing burden to world health and the economy (Devuyst and Pirson, 2007). In India ∼12% of the population suffers from KSD and females are more prone to the disease (Devuyst and Pirson, 2007; Alelign and Petros, 2018). KSD has a complex etiology comprising of both genetic, environmental, dietary factors. It also depends on nature or extent of pathogenicity of the mutation. If a mutation is both deleterious and homozygous or dominant, the likelihood of disease increases. As a result, age is a crucial characteristic to assess the extent of pathogenic mutation as because environmental impacts in disease penetrance requires time. (Goldfarb et al., 2005; Devuyst and Pirson, 2007; Guha et al., 2019). Most of the earlier studies on KSD were dealt only with adults and no documented evidence of pediatric KSD patients is available from India (Goldfarb et al., 2005; Prakash et al., 2019; Mitra et al., 2020). Moreover, we reported many genes responsible for Adults KSD patients in our earlier articles. CaSR, CLDN14, TRPV5, and CALCR are a few examples (Guha et al., 2015; Mitra et al., 2017; Guha et al., 2019; Mitra et al., 2020). Genetic screening is a useful tool to identify many underlying pathophysiology’s when there is a lack of proper clinical diagnosis. On the other hand, neonatal screening, referred to as new-born screening, is carried out shortly after a baby is born and assesses whether the kid is at risk of developing an illness. This allows early detection or prevention of various ailments. The type and timing of the genetic-screening methods are still up for debate across the country (Andermann and Blancquaert, 2010; Phadke et al., 2017). However, the rate of the incidence of Pediatric KSD is increasing rapidly throughout the world (Tasian et al., 2016; Issler et al., 2017). Therefore, In the present study we have reported Lower age (LA) NL patients for the first time in West Bengal, Eastern India.

Whole-exome sequencing (WES) offers a powerful technique to identify the monogenic as well as rare recessive genes (Halbritter et al., 2015; Daga et al., 2018; van der Ven et al., 2018; Suwinski et al., 2019). Here, we have conducted WES of 18 unrelated KSD patients, aged between newborn to 20 years (Daga et al., 2018) along with 20 adult KSD patients. Our study cohort showed the presence of Twenty-five mutations in eighteen genes from sixteen child KSD patients that were absent in the adult cases. Among those mutations, the most common mutations were in GRHPR, AGXT, and HOGA1 genes. Further, biochemical parameters such as serum creatinine, serum urea, serum calcium and urinary calcium were significantly higher in almost all child KSD patients. We also observed that lower age group patients developed hyperoxaluria, indicating the proneness to KSD. Additionally, mRNA expression by real-time quantitative PCR and protein expression by immunoblotting, along with protein activity and computational analysis established the role of c.494G>A GRHPR mutation in the development of KSD.

## 2 Materials and methods

### 2.1 Background survey and study participants

A Background survey using the patient’s database was conducted at the Department of Urology, Institute of Post-Graduate Medical Education and Research (IPGME and R) Kolkata from 2009 to 2016. Based on this survey the study population was divided into two age groups, 0–20 years as lower age or pediatric group (Hardin et al., 2017) and above 20 years representing adult age group (Daga et al., 2018). Study participants with any previous history of kidney stones between the ages of 1–20 years i.e., pediatric KSD patients were recruited when renal stones were identified by ultrasound, X-ray or multi-detector computed tomography (MDCT). Patients with any terminal disease were excluded from the study. Control subjects without any symptoms and no previous history of KSD were also included. The list of the collected LA samples is presented in Supplementary Table S1. The age of the control samples was above 18 years, and the sample was collected from April 2019 to March 2020, and from May 2021 to September 2021. This study was performed following principles of the Declaration of Helsinki with the ethical approval of the study protocol from the Ethics Committee of IPGME and R, Kolkata (Memo No. Inst/IEC/2015/436, dated 07/07/2017). An informed consent form was obtained from each study participant or their parent or legal guardian in case of children under 18.

### 2.2 Biochemical parameters

Serum concentrations of creatinine, calcium, urea, along with 24 h urine excretions of calcium, and phosphate were measured in both patients and healthy controls. Creatinine was estimated by using a modified Jaffe’s reaction (Delanghe and Speeckaert, 2011), while serum and urinary calcium were estimated by the arsenazo III method (Sepulveda, 2019). The ammonium molybdate method was used to quantify urinary phosphate (Goldfarb et al., 2005), and serum urea was estimated by the modified glutamate dehydrogenase method (Qin et al., 2013). All the above parameters were measured using the XL-600 Analyzer (Erba Mannheim, United States).

### 2.3 Characterization of kidney stones

The Surface morphology of Kidney stone samples (N = 5) was analyzed by Scanning Electron Microscopy (SEM; Model ZEISS EVO 18). The Elemental composition of samples was analyzed using energy disruptive spectroscopy or EDS (Hitachi S 3400N).

### 2.4 DNA extraction and whole exome sequencing

Genomic DNA was isolated from peripheral blood using DNA-QIAamp mini kit (Qiagen, Hilden, Germany) according to the manufacturer’s instructions. The Purity of DNA was determined in Varioskan Lux (Thermofisher). WES was carried out in the Illumina Hi-seq X10 sequencer. Sure select-XT Human All Exon V5+UTR kit from Agilent (Chen et al., 2015) was used for target enrichment. The generated paired end fast-q files of 150bp were analyzed for quality check, trimming, mapping, and annotation by CLC genomics workbench 21.0.4 using the biomedical genomics plugin. Final VCF files were analyzed further for variant prioritization.

### 2.5 Variant prioritization

VCF files were then subjected to IVA analyses (Qiagen, United States). Similarly, the data were revalidated with the application of various web servers such as mutation distiller (Hombach et al., 2019), phenolyzer (Yang et al., 2015) Moon (*Diploid-supporting Rare Disease Diagnostics*, n. d.) Wannovar (Yang and Wang, 2015). Pathogenicity of the mutations was calculated using polyphen 2, Wintervar (Li and Wang, 2017) following ACMG guidelines.

### 2.6 Sanger validation of *rs180177314*

Twenty-four (Hombach et al., 2019) cases or test samples and 51 control samples were genotyped for the rs180177314 of GRHPR gene. The PCR reaction mixture contained 50 ng genomic DNA, 0.5 mM forward and reverse primers, 5% DMSO, and 1x Green master mix (Promega). The PCR conditions were used according to our standardized methods. The primers for sanger sequencing is provided in (Table 5).

#### 2.6.1 Bioinformatics

Conservancy in genomic location of GRHPR gene was predicted using Phylop and phastcons Scores (Pollard et al., 2010; Hombach et al., 2019; Ramani et al., 2019). The stability of the mutated GRHPR protein structure was calculated in terms of RI and free energy change values (DDG) by the I-Mutant 2.0 tool (Capriotti et al., 2005). Then, the evolutionary conservancy of mutant amino-acid residue was determined using the Con-Surf web server (Landau et al., 2005). Moreover, HOPE ((Venselaar et al., 2010)), phyre2 (Kelley et al., 2015), and missence3d (Khanna et al., 2021) online tools were used to analyze the 3D structure of mutated GRHPR protein in terms of clashes, Rotamers, charge, and site detection. 3D models for the mutant protein were generated by replacing wild amino acid residue with the mutated residue of the native sequence of the templates 2GCG using I-tasser (Roy et al., 2010) and the Swiss model (Waterhouse et al., 2018). Further, Structural similarities between native and mutant models were investigated based on C-score, TM-score, and RMSD scores. Furthermore, 3D structures of the protein were analyzed by SWISS-MODEL (36). Afterward, mutant protein vs. wild protein was subjected to docking using a web server of patch-dock (Duhovny et al., 2002; Schneidman-Duhovny et al., 2005) and followed by fire-dock (Mashiach et al., 2008). The docking was viewed and analyzed in Discovery studio (Biovia, 2022).

#### 2.6.2 PBMC isolation

GRHPR mutation c.494G>A was found among the cohort, we retraced and found only four willing test samples from the previous collection and 2 from the new collection with the same mutation. And others were untraceable due to long COVID-19 lockdowns. Fresh peripheral blood samples were collected in EDTA coated vial. Then, blood was diluted with phosphate buffer saline (PBS) in a 1:1 ratio in a separate polypropylene tube. The diluted blood sample was added in a separate tube with histopaque®-1077 in a 2:1 ratio. The entire mixture was then centrifuged in 400 g for 35 min at 37°C. A foggy phase of PBMC is observed in between precipitated RBC and an yellowish upper phase. The middle foggy layer was Pipetted out and stored at −80°C for further use.

#### 2.6.3 Real-time quantitative PCR

To evaluate the effects of c.494G>A mutation in GRHPR mRNA expression, we have isolated the total RNA from monocytes of both test (N = 6) and control (N = 6) samples following standard TRIzol methods (Simms et al., 1993)*.* Then cDNA was synthesized from isolated RNA using the High-Capacity cDNA Reverse Transcription Kit (Thermofisher Scientific) following the manufacturer’s protocol. The reaction was performed using our standardized methods. The transcript level of all the genes was normalized with an internal reference, the human glyceraldehyde 3-phosphate dehydrogenase (GAPDH) gene. The relative expression ratio of each gene was calculated using the comparative ΔΔ*C*_T_ value as described previously (Schmittgen and Livak, 2008). All the primers used in this study are listed in (Table 1).

**TABLE 1:** Primer for RTqPCR.

GRHPR	Forward primer 5′-CAG​ATG​TCC​TGA​CAG​ATA​CCA​C -3′
	Reverse primer 5′-GCC​ACC​ATT​CTT​CAC​TTC​CT-3′
GAPDH	Forward primer 5′-CTG​CAC​CAC​CAA​CTG​CTT​A -3′
	Reverse primer 5′- GTC​ATG​AGT​CCT​TCC​ACG​ATA​C-3′

#### 2.6.4 Immunoblotting

Cell lysates from Human test (N = 6) samples and Control (N = 6) were prepared using RIPA buffer containing protease and phosphatase inhibitors (Sigma). An equal amount of protein was loaded to each well in SDS-PAGE (10%) and immunoblotting was carried out using standard techniques (Mahmood and Yang, 2012). Primary antibodies including anti-GRHPR antibody TA502091, anti-GAPDH antibody ab8245 were used. Immunoblots were developed by the Chemiluminescence method using Clarity Western ECL substrate **(**Bio-Rad). Densitometric quantification of Western blots was performed by utilizing ImageJ software (NIH). GRHPR protein expression was normalized to loading controls GAPDH and expressed relative to fold change (Neris et al., 2021).

#### 2.6.5 Immunocytochemistry

Isolated PBMC from the test and control human samples were placed in 35 mm culture dishes and incubated for 3 h in complete media (DMEM with10% FBS) for cell attachment. The cells were then rinsed three times with ice-cold PBS and fixed using methanol. The cells are then prepared for immunocytochemistry using a standard protocol (Polak and van Noorden, 1983) using an anti-GRHPR antibody. Cells incubated with Alexa fluor 555 tagged Anti-Mouse secondary antibody (Thermo Fisher A32727) and counterstained with DAPI. The final image was captured using the Floyd cell imaging system (Thermo-Fisher).

#### 2.6.6 GRHPR activity

Herein, we determined the GRHPR activity (glyoxylate reductase) assay in (*n* = 12) of 6 KSD patients and 10 normal individuals) by measuring the rate of formation NADP in the reaction mixture. The reaction mixture contained the substrate (glyoxylic acid) at a concentration ranging from 0.2 to 2.5 mm, NADPH (0.35 µm), isolated protein (0.01 mg/ml) from PBMC of both case and control samples in PBS at 37°C, P^H^ 8. The formation of NADP was measured at 260 nm (Fruscione et al., 2008; Harris and Keshwani, 2009). Each experiment was in triplicate. Calculations were performed using GraphPad Prism (San Diego, California, United States).

## 3 Results

**FIGURE 1:**
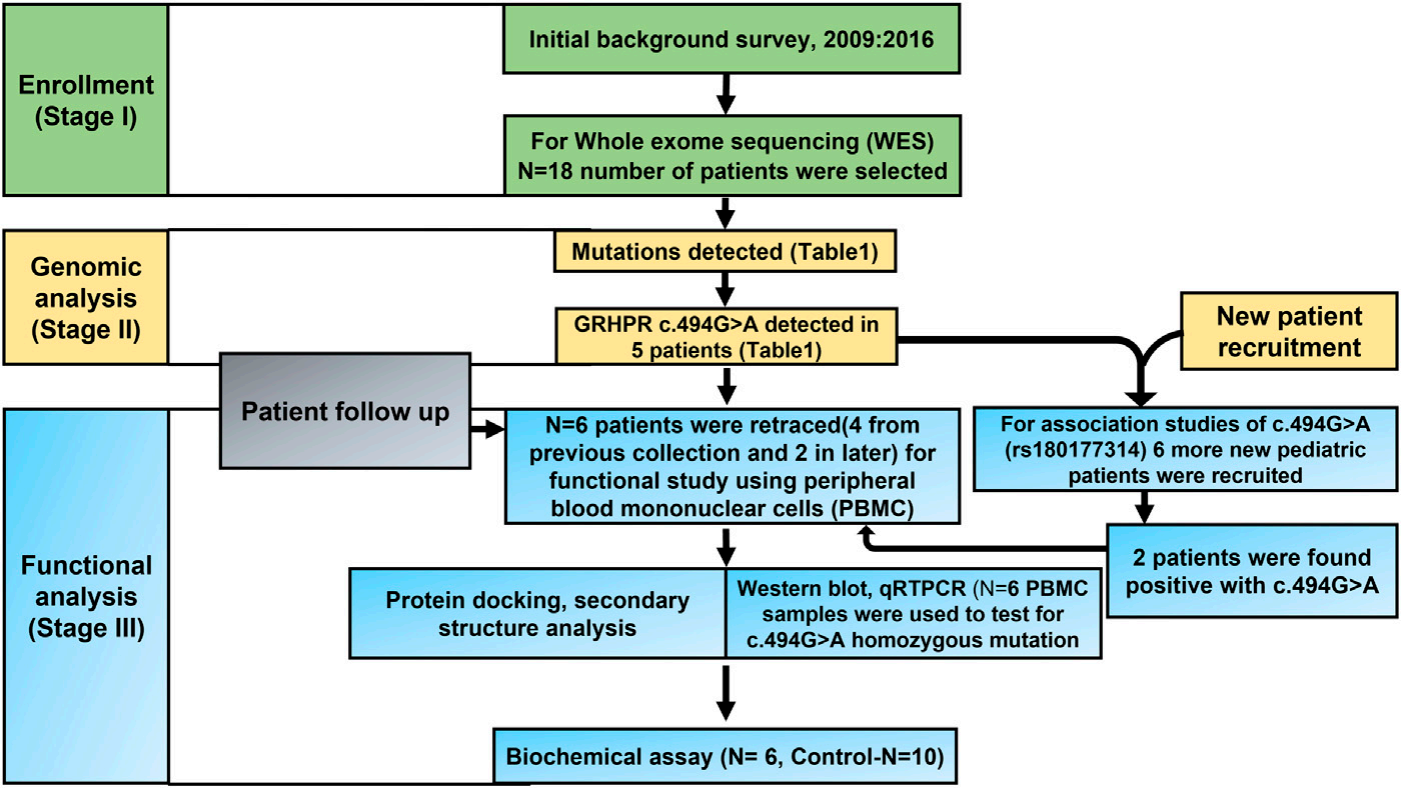
Consort diagram of the Study design.

**FIGURE 2:**
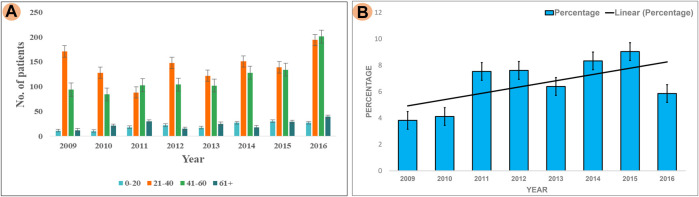
Analysis of Survey; **(A)** Showing prevalence of KSD in all the age groups (0–20, 21–40, 41–60, 60+) years per year, between 2009–2016 **(B)** Percentage of affected lower age group (0–20) KSD patients per year, between 2009 and 2016.

The results of our survey data showed that the KSD prevalence in lower age group is <10% which is significantly lesser (*p* < 0.05), compared to the adult age group (Figure 1 and Figure 2). Moreover, in the lower age group disease prognosis was observed at an early stage of life, indicating a high probability of genetic factors for the causation of KSD (Langman, 2018). So, we performed WES in 18 individuals of the lower age group of unrelated families with KSD. All affected individuals had hypercalciuria or hyperoxaluria together with KSD (Supplementary Table S1). Among them, 16 patients showed genetic mutations related to KSD or NL (Table 2). Out of which 8 patients had pathogenic mutation in the genes related to hyperoxaluria causing calcium oxalate stone. Only 2 individuals among the patients showed no mutation related to KSD.

**TABLE 2:** Mutation detected in all the pediatric samples with KSD or NL.

Sl. No	Tools	Gene name	Present in cases	Zygosity	Pos (grch37)	rs number	Change in DNA	Ref seq	Amino acid change	Novelty	pph2	ACMG	Predictions
1	Moon-diploid, Mutation Distiller, Wintervar, Phenolyzer	GRHPR	KS1, S4, KS10, KS12, KS14	HOM	9:37,429,729	rs180177314 MZ826703	G>A	NM_012203	G165D	-	1	PM1, PM2, PP3, PP5, PP6	Damaging/pathogenic
2	Do	SERPINH1	KS1	HET	11:75277687	rs541595707	G>A	NM_001235	S98N	-	0	PM1, PM2, PP2	Benign/ Uncertain significance
3	Do	AGXT	S4	comp HET	2:241808588	rs180177180	T>A	NM_000030	I56N splicing impaired	-	1	PM2, PP3, PP5	Probably damaging/ Uncertain significance
4	Do	AGXT	KS4	HOM (frameshift)	2:241817038	rs398122322	c.33dupC	NM_000030	p. Lys12fs	-	NA	-	Uncertain significance
5	Do	AGXT	KS6	HET	2:241808325	Novel MW084974	C>T	NM_000030	L15F	Yes	NA	PM2, BP4	Uncertain significance
6	Do	FBN1	KS6, KS5	HET	15:48782174	rs112287730	C>T	NM_000138	A986T	-	0.458	PM2, BP3, BS2, BP1	Possibly damaging/ Likely benign
7	Do	FBN1	KS4	HET	15:48764757	Novel OP328168	C>T	NM_000138	A1443T	Yes	0.316	PM2, BP1	Benign/ Uncertain significance
8	Do	HSPG2	KS1	HET	1:22175394	rs758731100	C>A	NM_005529	G2526V	Yes	0.022	PM2, PP2	Benign/Uncertain significance
9	Do	HSPG2	KS5	HET	1:22191473	rs138460117	A>T	NM_005529	F1497I	-	0.997	PP2, BS1, BS2	Probably damaging/ Uncertain significance
10	Do	ACVRL1	KS1	HET	12:52307462	Novel OP328167	C>T	NM_000020	R145W	Yes	0.001	PM1	Benign/Uncertain significance
11	Do	ATP6V1B1	S4, KS1	HET	2:71192103	rs142905621	G>A	NM_001692	R465H	-	0.997	PM1, PP3,, BS2, BP6	Probably damaging/ Likely Benign
12	Do	MOCS1	S4	HET	6:39893505	rs751538238	C>T	NM_005943	R112Q	-	0.131	PM2	Benign/Uncertain significance
13	Do	CRTAP	KS6	HET	3:33155657	rs553076085	C>T	NM_006371	R30C	-	0.999	PM1, PM2, PP3, BS2, BP1	Probably damaging/ Likely benign
14	Do	HOGA1	KS6	HET	10:99361647	Novel OP328164	T>A	NM_138413	V245D	Yes	1	PM1, PM2, PP3, BP1	Probably damaging/ Uncertain significance
15	Do	HOGA1	S13	HET	10:99361747	rs770050262	G>A	NM_001134670	A115A	-	0	PP3, PP5	Benign/ Uncertain significance
16	Do	USP8	KS7	HET	15:50769490	Novel OP328166	T>C	NM_005154	S338P	Yes	0.997	PM1, PM2	Probably damaging/ uncertain significance
17	Do	CACNA1D	KS2	HET	3:53777084	rs567068933	A>G	NM_001128840	N953S	-	0.666	PM1, PM2, PP3	Possibly damaging
18	Do	COL1A2	KS12	HET	7:94045747	Novel OP259503	G>A	NM_000089	A599T	Yes	0.891	PM1, PM2, BP3, BS1	possibly/ Uncertain significance
19	Do	COL1A2	KS2, K15	HET	7:94037534	rs375401215	G>A	NM_000089	A227T	-	0.181	PM1, PM2, PP3,BS2,BP6	Benign/ Likely benign
20	Do	VIPAS39	KS3	HET	14:77910662	rs372813446	C>T	NM_001193315	R176H	-	1	PM1, PM2, BP1	Probably damaging/ Uncertain significance
21	Do	ATP7B	KS11	HET	13:52508984	rs148081616	C>T	NM00053	D1229N	-	0.999	PM1, PM2	Probably damaging/ Uncertain significance
22	Do	ATP6V1B2	KS12	HET	8:20062019	rs200124277	A>G	NM001693	N54S	--	0.037	PM1, PM2, BS2	Benign/Uncertain significance
23	Do	CLIP2	KS14	HET	7:73753191	rs61739991	C>T	NM0038	P179S	-	0.024	PM1, BP6	Benign/ Uncertain significance
24	Do	VDR	KS15	HET	12:48258947	Novel OP328165	G>A	NM000376	R54W	Yes	1	PM1, PM2, PP2,PP3	Probably damaging/ Likely pathogenic
25	Do	SLC34A1	KS8	HET	2/44502889	rs765538592	T>C	NM_000341	V72A	-	1	PM1, PM2, PP3	Probably damaging/ Uncertain significance

WES data identified the most frequent mutation in three genes that are associated with hyperoxaluria (Table 2). A single GRHPR mutation (rs180177314 G>A) with homozygous conditions was found in 30% of the recruited patients of lower age group. Recessive or causative mutations were detected in 18 genes (Table 2), while damaging mutations were detected in 13 genes. Among 16 individuals 5 were GRHPR, 3 individuals were AGXT, 2 was HOGA1, and others were from SERPINH1, ACVRL1, MOCS1, USP8, COL1A2, VIPS39, ATP7B, ATP6V1B1, FBN1, HSPG2, CRTAP, VDR, and SLC34A1. This well depicted using a pictorial graph in Supplementary Figure S3 and Supplementary Table S2 The family history, status of consanguinity, and detailed phenotype of individuals are shown in Supplementary Table S1. Eight patients showed causative mutations for hyperoxaluria, and 3 detected mutations were novel pathogenic variants (Table 2). Nucleotide variant rs180177314 (G>A) in homozygous condition was functionally analyzed for the first time from human PBMC samples (accession no. MZ826703). Moreover, a SNV rs141428607 G>T of the SLC25A5 gene variant was found in 16 samples of the 18 studied patients. Further, String DB showed the interaction of SLC25A5 (Calvo et al., 2016)with the GRHPR gene (Supplementary Figure S4).

### 3.1 Biochemical analysis

The results showed that blood urea, creatinine, serum calcium, and urinary calcium were significantly higher (Table 3) in patients having a mutation in any of the 3 hyperoxaluria genes, than those of both control and adult kidney stones.

**TABLE 3:** Biochemical status of patients with Hyperoxaluria (*p* < 0.05, denoted in italic along with the ‘*p*’ of *p*-value (as per convention) †At diagnosis; SD = Standard deviation).

Characters	Cases with hyperoxaluria (N = 10)	Control (N = 85)	*p*-value (hyperoxaluria vs. control)	Other KSD cases (95)	*p*-value (hyperoxaluria vs. other KSD)
Blood urea (mg/dl)	56 ± 44.3	27.93 ± 7.023	0.00002	30.48 ± 13.83	0.0002
Serum creatinine (mg/dl)	2.36 ± 3.6	1.22 ± 1.3	0.0002	1.244 ± 0.72	0.00000001
Serum calcium (mg/day)	10.325 ± 1.7	9.66 ± 0.50	0.025	9.51 ± 0.50	0.002
24-h urinary calcium (mmol/day)	48.9 ± 15.7	6.13 ± 3.89	5.48 e−27	8.79 ± 5.90	7.26 e−20

### 3.2 GRHPR G165D study

#### 3.2.1 Statistical analysis of *rs180177314*

We have calculated the odds ratio (95%CI, *p* < 0.05) for the risk allele A in the study cohort (Altman, 1991). For the variant (rs180177314), the A allele frequency distribution in the patient population (0.29), compared to controls (0.059%). Our result indicates an increased risk of 6.59 in disease progression in individuals carrying the A allele (Table 4).

**TABLE 4:** Allele frequencies recorded in controls and patient samples.

SNPs	Allele	Allele frequency		Odds ratio (95%CI)	*p*-Value
*rs180177314*		Case (24)	Control (51)		0.0114
	G	0.71	0.94	6.59 (1.528–28.50)	
	A	0.29	0.06		

#### 3.2.2 Conserved site (GRHPR G165D) analysis by bioinformatics

**FIGURE 3:**
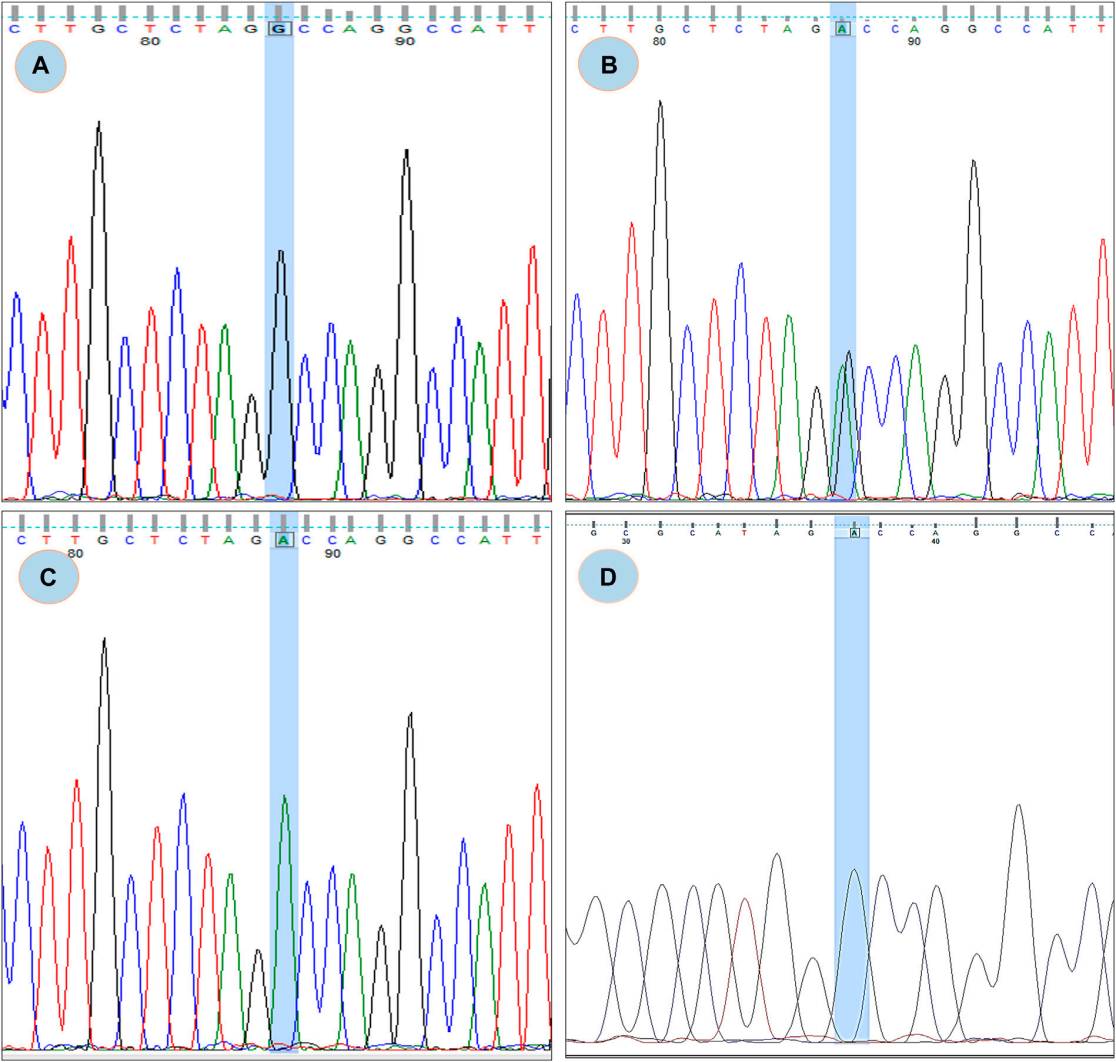
Chromatogram of rs180177314; **(A)** showing GG genotype, **(B)** showing GA genotype, **(C)** showing AA genotype **(D)** Chromatogram from CDNA sequence showing no sign of splicing error.

**FIGURE 4:**
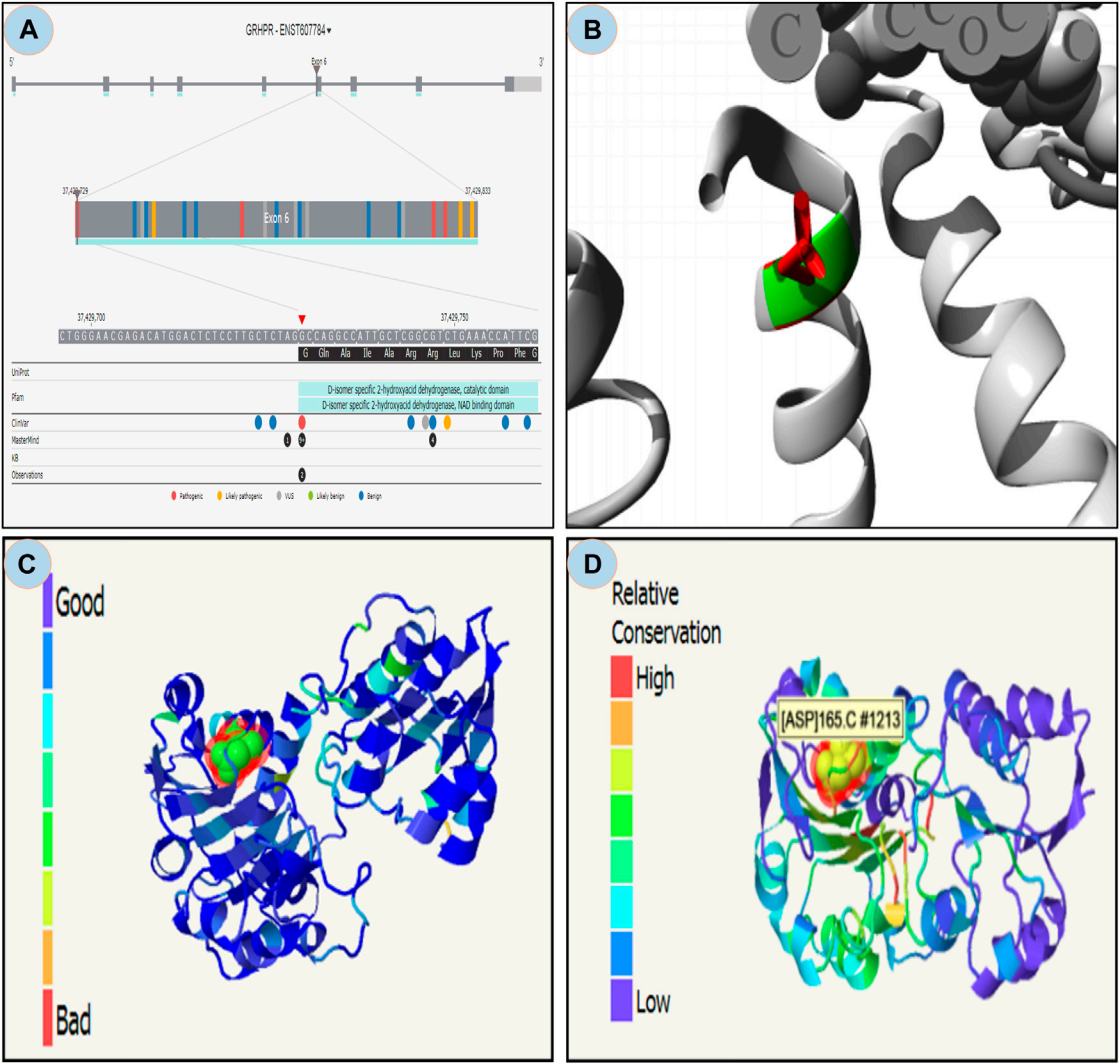
Moon diploid: **(A)** Showing mutation in the region of NAD binding domain; Hope: **(B)** Mutated residue (ASP) shown in red is large compared to wild residue in green; **(C)** Phyre: showing relatively high clash (left bar indicates gradient of clash) in the mutated region; and **(D)** Showing relative high conservation in the mutated region.

From the study it was found that the phylop and phastcons scored positive value of 5.408 and 0.832 respectively, which indicates location g.37429732G>A in the genomic sequence is highly conserved and expected slower evolutionary change. Also, the Consurf server predicted a high conversed site in 165 residues in GRHPR protein. Moreover, The Project HOPE server, Missence3d, and phyre2 revealed that the mutant residue ASP (aspartic acid) is of bigger sizes (Figure 3 and Figure 4B), present in the conserved region (Figure 4D) and causes clashes (Figure 4C) than the wild-type residue Gly (glycine); and these variations in size and hydrophobicity can disrupt the H-bond interactions with the adjacent molecules. Furthermore, I-tasser and Moon diploid server predicted that the location of the mutation is very close to the NAD binding domain (Figure 4A) and can disrupt its function.

#### 3.2.3 Docking analysis

**FIGURE 5:**
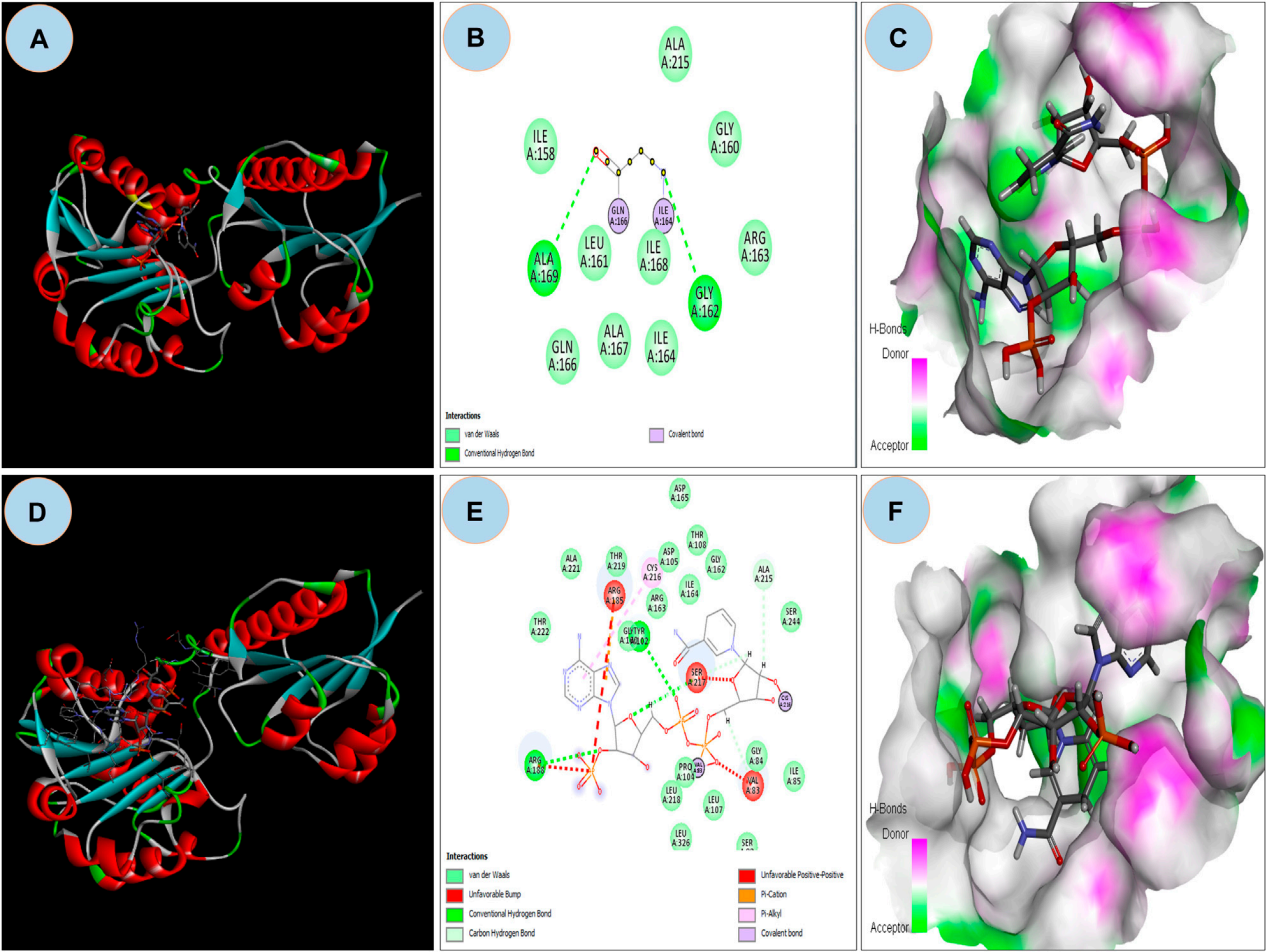
**(A)** Showing docking with NDP ligand on NAD binding domain on Wild GRHPR protein in 3D; **(B)** showing interactions of wild GRHPR protein with NDP ligand in 2D; **(C)** Enlarged view of the Receptor site of Wild GRHPR protein vs. NDP ligand wrt to H-bond pocket; **(D)** Showing docking with NDP ligand on NAD binding domain on Mutated GRHPR protein in 2D; **(E)** 2D image showing unfavorable bump in the mutated residue, here shown in red which missing in wild in NAD site, **(F)** Enlarged view of the receptor site shows H-bond pocket in mutated GRHPR protein vs. NDP ligand.

The ASP residue causes an unfavorable bump and steric collision in the NAD binding domain that overlaps and affects stability (Figure 5A and Figure 5B) between non-bonding atoms (Ramachandran et al., 2011). The PatchDock score states, more the steric clashes lower the score (Fruscione et al., 2008). PatchDock predicted a score of 5782 of the mutant protein, which is lower than the score 5822 of wild protein. In the NAD binding region, FireDock predicted global energy of -9.04 in mutant protein and -41.82 in wild type; this value is related to free binding energy, with a smaller negative value implying a bigger free binding energy.

#### 3.2.4 GRHPR mRNA expression analysis by using RT-qPCR

**FIGURE 6:**
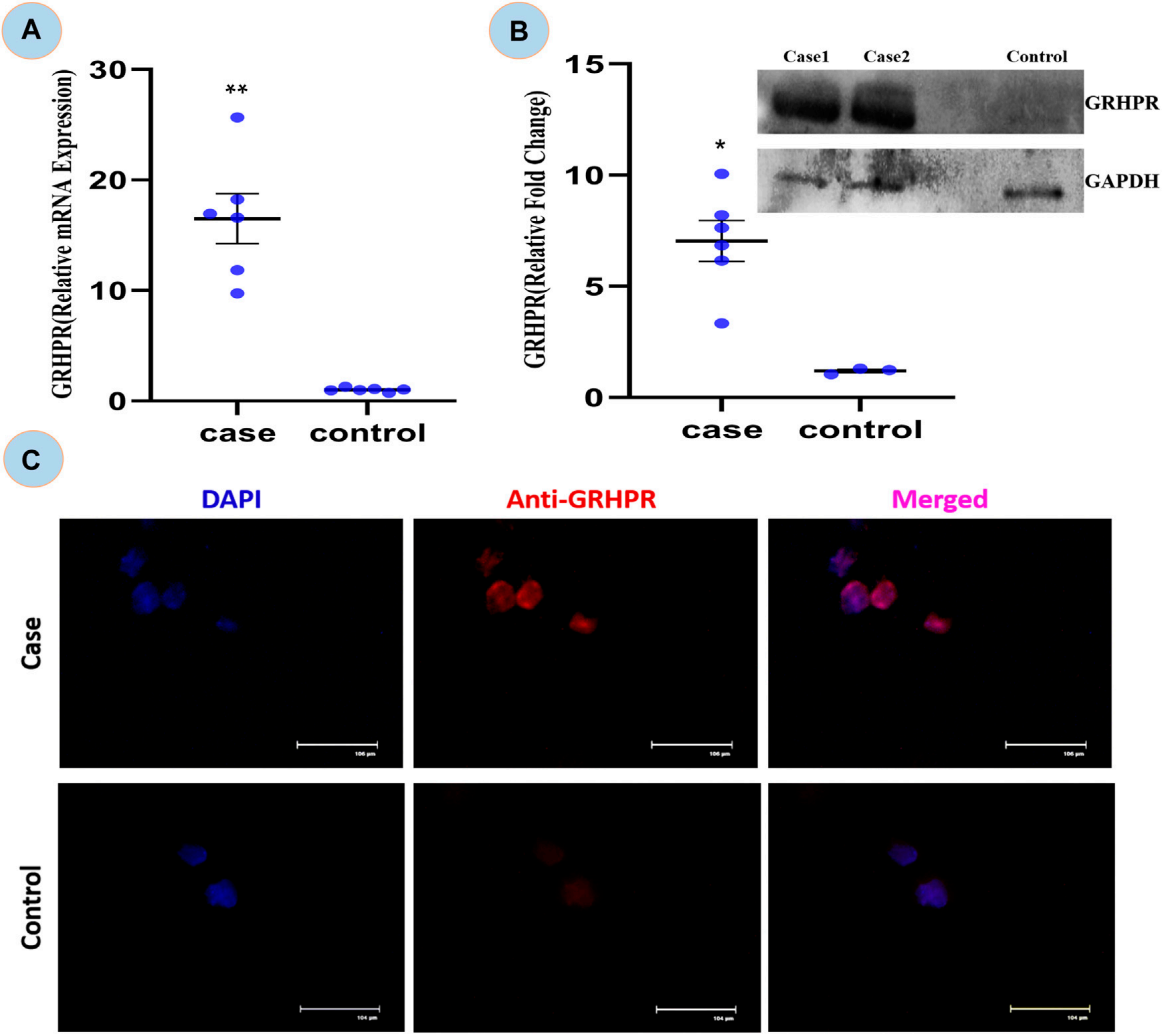
**(A)** Relative mRNA expression of GRHPR gene in test (C.494G>A) Vs. Control, (N = 6); **(B)** Immunoblot showing protein fold change wrt GRHPR gene in Case (N = 6) VS. Control; **(C)** Immunocytochemistry (ICC); showing monocytes of Case and control. *p*-value less than 0.05 is represented by * and less than 0.01 **.

The expression of GRHPR gene of c.494G>A mutation was significantly greater (*p* < 0.01) in test PBMC samples than that of the control (Figure 6A). The RT-qPCR data showed the upregulation of GRHPR gene expression, ∼16 folds in test cases. So, we further investigated the GRHPR protein expression among the study samples.

#### 3.2.5 Determination of GRHPR level by immunoblot

The GRHPR protein was upregulated by ∼7-fold in samples with mutations (Figure 6B). It indicated that the proteins were upregulated to compensate for defective enzymes due to mutation. So, there is a much alike higher level of mRNA and protein expression of GRHPR gene in the test samples when compared to the control group.

#### 3.2.6 Immunocytochemistry from patients’ PBMC

The ICC is concerned the transient signal of the anti-GRHPR Ab in control PBMC expression demonstrated only a limited amount of GRHPR expression (Figure 6C). Whereas Patients’ PBMC samples, on the other hand, had strong GRHPR expression, reaffirming the findings of the immunoblot.

#### 3.2.7 Determination of GRHPR enzyme activity by biochemical assay

**FIGURE 7:**
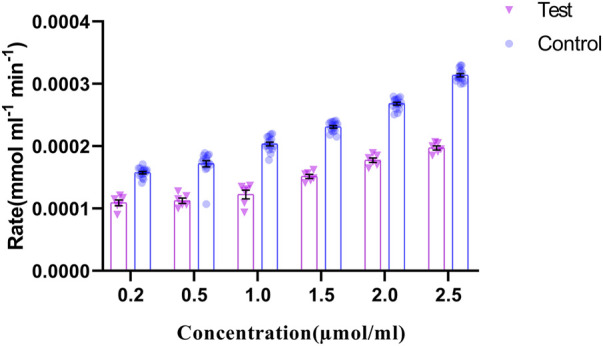
GRHPR Enzyme activity in the presence of different substrate (Glyoxylate) concentration (µmol/ml) between test (C.494G>A) (N = 6) and control samples (N = 10); and the rate of NADP formation demonstrated as mmol ml^−1^ min^−1^.

The regulation of *p*. G165D GRHPR in hyperoxaluria patients, could be linked with its enzymatic activity, while the rate of product formation was determined by measuring NADP at 260 nm. Our data revealed that there were significant changes (*p* < 0.05) in the enzyme activity between case and control samples (Figure 7).

## 4 Discussion

This study summarizes on age group stratification of West Bengal in the eastern Indian population, with a focus on LA patients suffering with NL. We observed a significant difference in the number of individuals between lower age KSD patients and adult KSD patients. Among our collected samples a higher number (30%, 5 out of 18) of GRHPR mutations (rs180177314, G>A) were found in the LA patients. Population frequency showed that the ‘G' allele and the ‘A' allele frequencies among control individuals were 0.94 and 0.06, respectively (Table 4), which matches the allele frequency in the Ensembl database from the South Asian population (‘G' allele 0.99 and ‘A' allele 0.01). With association study we have found that individuals with the “A" allele had a 6.59-fold higher risk of kidney stone development than those with the “G" allele, with a 95% confidence interval (CI) of 1.52–28.50. As a result, the allelic shift from G to A for the GRHPR gene, rs180177314, can be anticipated as a risk factor for kidney stone formation and hyperoxaluria.

PhyloP and PhastCons scores verified that the location of rs180177314 is evolutionarily conserved and mutation c.494G>A in that location changes the amino acid residue from glycine to aspartic acid (Buried charge) (Ittisoponpisan et al., 2019). The mutated residue introduced steric hindrance, clashes and conflicts which resulted in an unstable NAD binding site. The translated protein from this NAD binding site may then contribute to the impairment of GRHPR enzyme. (Figure 4A). The function of the enzyme is to maintain the cytosolic concentration of hydroxy pyruvate and glyoxylate at a very low level; thus, preventing the formation of oxalate (Cramer et al., 1999). The mutation in the GRHPR gene may also be the responsible factor for its instability, alteration of hydrogen bonding pattern, and conformational change. Moreover, our docking analysis showed that the mutated residue in the GRHPR protein impaired its free binding energy.

Expression studies from the case samples delineated that the mutation in the enzyme might be responsible for the up regulation of the GRHPR gene in both experiments. On the other hand, loss of enzyme activity of the GRHPR gene due to a particular mutation at conserved site suggests a loss of function despite gene upregulation (Vockley et al., 1996). The deficiency of glyoxylate reductase activity presumably causes the impaired conversion of glyoxylate to glycolate and can induce oxalate formation (Cramer et al., 1999). SEM and EDX analysis of stones revealed characteristics of whewellite stones Supplementary Figure S1C in Hyperoxaluria patients, which is quite natural, and dent of white patch can also be shown in our study Supplementary Figure S1CB. Furthermore, the SEM-EDS studies revealed an increase in the proportion of calcium (Ca) weight in the stones of patients with Hyperoxaluria Supplementary Figure-S2B and a peak of Ca with hyperoxaluria. (Racek et al., 2019). We found out that mutation C.494 G>A in the GRHPR gene might be the reason for the impairment of dual enzyme activity i.e., Glyoxylate to glycolate reduction and its reconversion to glyoxylate again, and the conversion into glycerate to hydroxy pyruvate. Also, from the enzyme activity analysis, we have noted the decreased enzymatic activity of glyoxylate reductase in GRHPRc.494G>A patients (Figure 7) probably due to the loss of function mutation in GRHPR gene.

The exome analysis revealed that other disease-causing mutations related to hyperoxaluria were also present in AGXT (PH1), and HOGA1 (PH3). Moreover, the pathogenic mutation in the GRHPR is intriguing in the context of age range and homozygous condition that might account for the severity of the disease In addition, all of the other disease-causing mutations found in the cohort with nephrolithiasis are presented in Supplementary Table S2 along with Table 5 their putative disease designation. Furthermore, Previous study reports analysed that PH1(AGXT) is the most common subtype (80%) and often is associated with poor clinical outcome. Both PH2 (GRHPR) and PH3 (HOGA1) are deemed to be extremely rare but recent studies showed that PH2 and PH3 are underdiagnosed specifically in KSD prone zone.

**TABLE 5:** Primers for sanger validation of rs180177314 of GRHPR gene.

**rs180177314**	Forward primer 5′- CGGGCTGTGCTGATGAAA -3′
	Reverse primer 5′-CAG​ATA​GGC​TCC​TGT​GGA​AAT​C-3′

It has been found that PH2 investigation in many cases were underdiagnosed or overlooked (Webster et al., 2000; Rumsby and Hulton, 2017). PH2 is a rare inherited and rare monogenic disease. In case of PH2, oxalate and l-glycerate excretion were found to elevate because of the impairment of GR and HPR activities. We have also showed the impaired GR activity in context of diminished NADP production in KSD patients compare to control (Figure–7). Furthermore, it should be mentioned here that with the excretory oxalate level, primary hyperoxaluria type cannot be determined properly. Fallacy is that PH2 patients might be more prevailing than expected previously. Indeed, according to Mayo Clinic, PH2 patients were misdiagnosed with PH1 (Gagnadoux and Broyer, 1995; Chlebeck et al., 1994). Higher degree of disease-causing mutation in GRHPR may indicate low number of patients studied (Cregeen et al., 2003)The impairment of GRHPR activity in monocytes may be a sign of PH2 (Takayama et al., 2014) and further genetic analysis can also conform the disease, which we uncovered in our research work. Takayaman et al. also showed that among 45 patients with PH2, 9 individuals with GRHPR mutation (c.494G>A) were from Indian subcontinent. As such prevalence of PH2 is higher than previously thought as demonstrated (Takayama et al., 2014).

Interestingly, it has been reported that genotype-phenotype correlation is an intricate issue in PH1 patients and it may be due to the environmental factors and other modifier gene (Beck and Hoppe, 2006; Hopp et al., 2015). It has been found that PH3 is less severe and slowly progressive than PH1 and PH2. Our findings through NGS and sanger ((Williams et al., 2015)) also deciphered that all the GRHPR mutation (c.494G>A) were in child onset <12 years (Supplementary Figure S3 and Supplementary Table S1) and the same mutation was not present in adult KSD patients. So, age-range, level of calcium, monocyte assay may be compared with genotype (specific mutation) but the study should be widened to remark a specific conclusion.

Also, the underlying cause of missense mutation in SLC25A5 detected in our study, may have some tie-up with GRHPR; that is yet to be studied. Also, SLC25A5 has a role in the calcium homeostasis (Lytovchenko and Kunji, 2017) but its relation to hyperoxaluria needs to be investigated.

## 5 Conclusion

Our study divulged the underlying cause of kidney stones in the lower age group. WES analysis showed lower age group patients are prone to hyperoxaluria and harbor a predominant mutation c.494G>A in the GRHPR gene. Functional and biochemical studies backed the upregulation of the gene with impaired enzyme activity due to the mutation. Therefore, GRHPR c.494G>A is an important potential marker and monogenic cause of lower age KSD in the studied population.

Study Limitation: The study is limited to the population of West Bengal (Eastern India).

## Data Availability

The datasets presented in this study can be found in online repositories. The names of the repository/repositories and accession number(s) can be found below: Gene name: AGXT, accession number: MW084974. Gene name: GRHPR, accession number: MZ826703. Gene name: COL1A2, accession number OP259503. Gene name: ACVRL1, accession number OP328167. Gene name: USP8, accession number OP328166. Gene name: VDR, accession number OP328165. Gene name: FBN1, accession number OP328168. Gene name: HOGA1, accession number OP328164.
