# Supplementary material for: Homozygous GRHPR C.494G>A mutation is deleterious that causes early onset of nephrolithiasis in West Bengal, India
Source: Front Mol Biosci. 2022 Dec 22;9:1049620. doi: 10.3389/fmolb.2022.1049620 (PMC9815608; doi:10.3389/fmolb.2022.1049620)
Supplement: Supplementary file 1 [file Table1.DOCX]

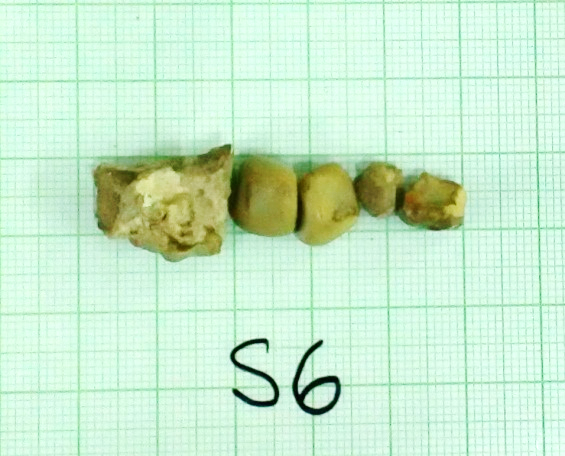

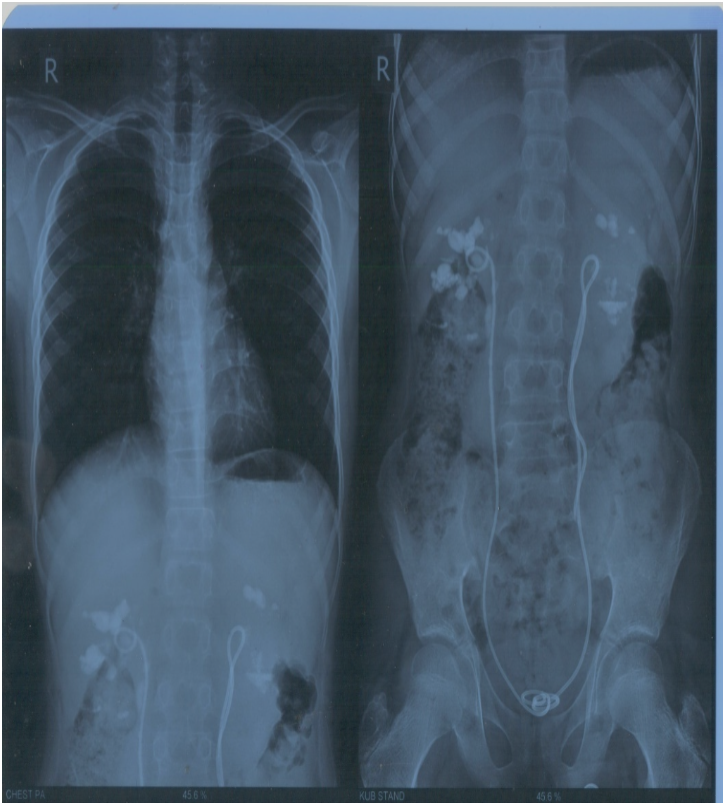


(a)

(b)


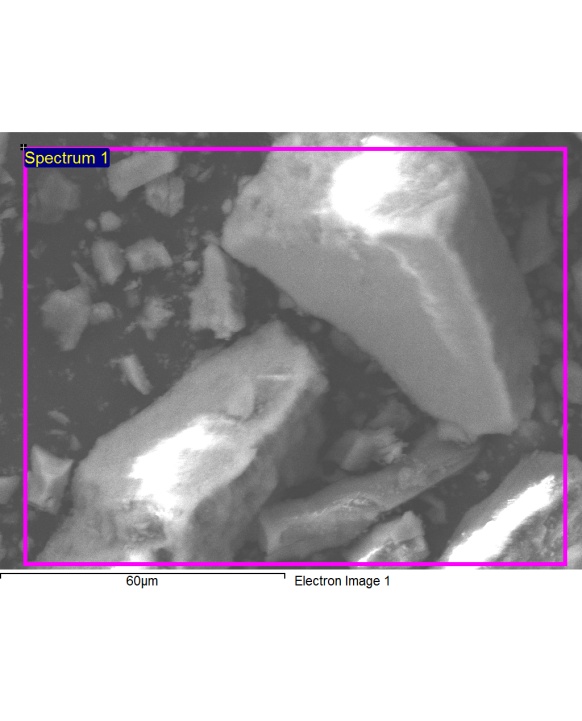


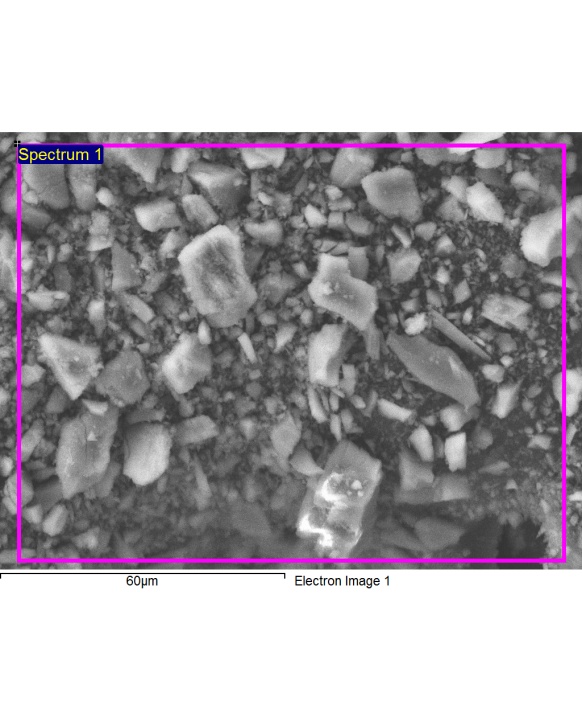


(d)

(c)

Supplemetary Figure1: (a) X-ray image of showing kidney stones with GRHPR mutation in KSD patient; (b) Whewellite urinary stones, made up of characteristics crystals indicating the hyperoxaluria(Cloutier et al., 2015; Daudon et al., 2008; Singh & Rai, n.d.) (c) The morphology and internal structure of whewellite kidney stones showing whewellite stones, formed due to primary hyperoxaluria type some unique characteristics, such as an unorganized internal structure and the presence of tabular crystals and crystal aggregates.


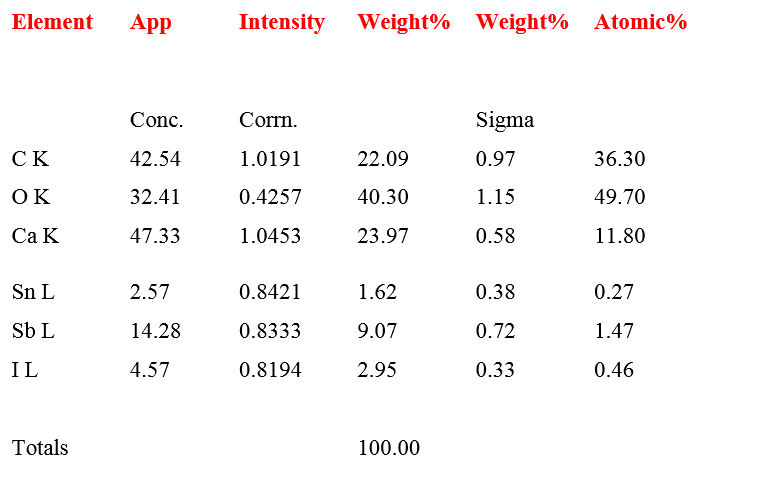

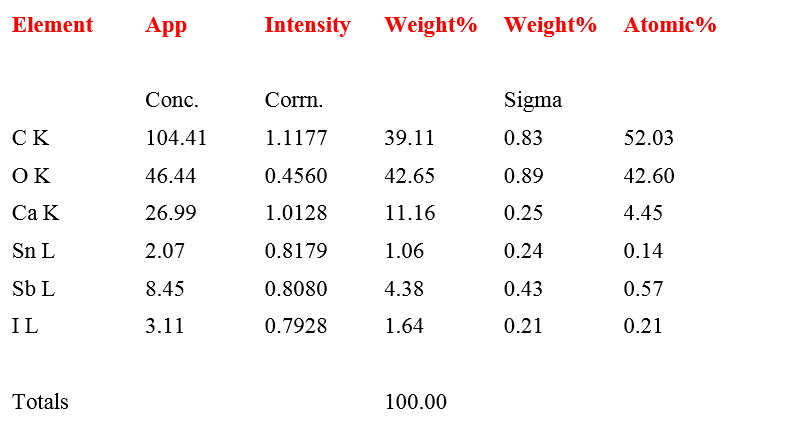

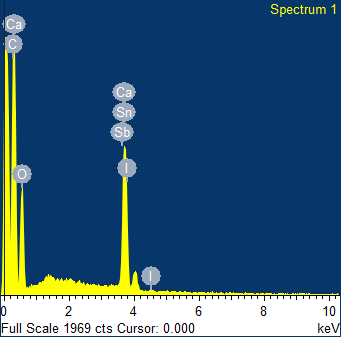

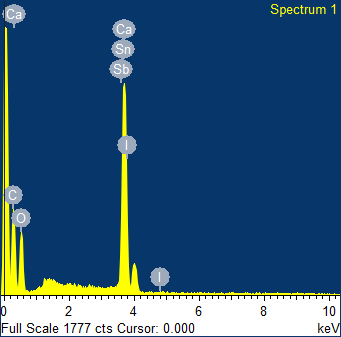


(a)

(b)

Supplementary figure 2 . In EDX spectra of kidney stones, a sharp wide peak was observed between the 3–4 keV spectral region. The incident of that sturdy signal from Ca, O, and C atoms (23.97, 40.30, 22.09) percentage respectively specified that the kidney stone was dominated by calcium oxalate. However, there was other peak of EDX for Sb and Sn, signifying that they were mixed precipitates. (a) showing weight percentage of calcium in normal kidney stone vs (b) weight percentage of Kidney stone with GRHPR mutation.(Racek et al., 2019)


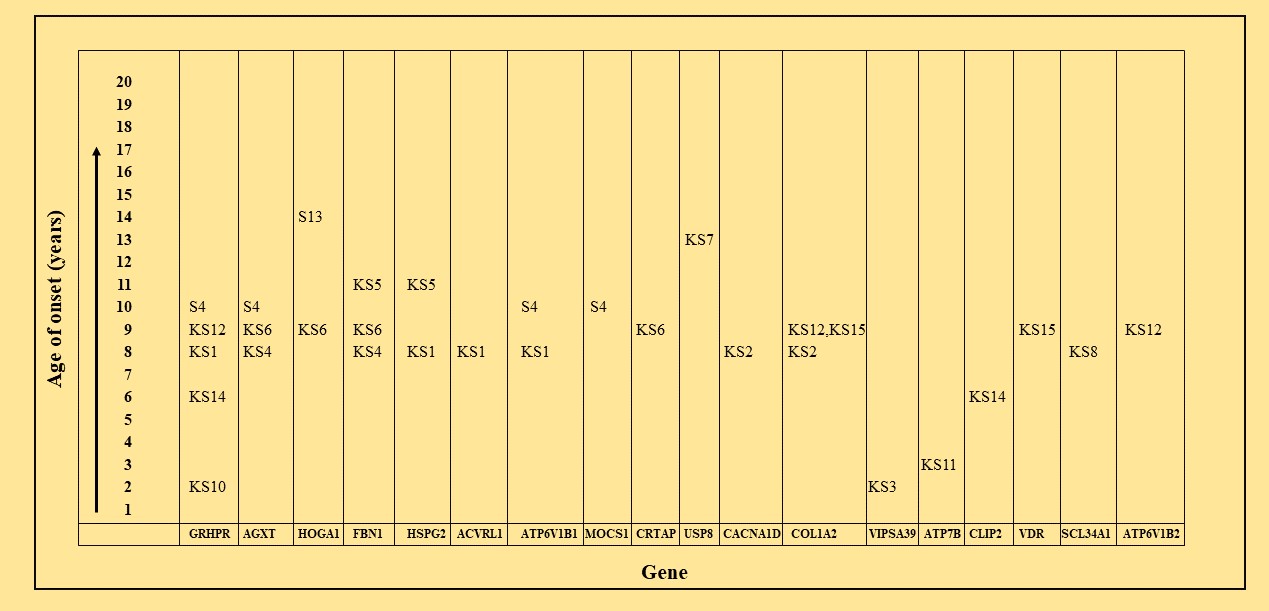


Supplementary Figure 3. Figure showing all the genes found in respective sample along with their age of onset.


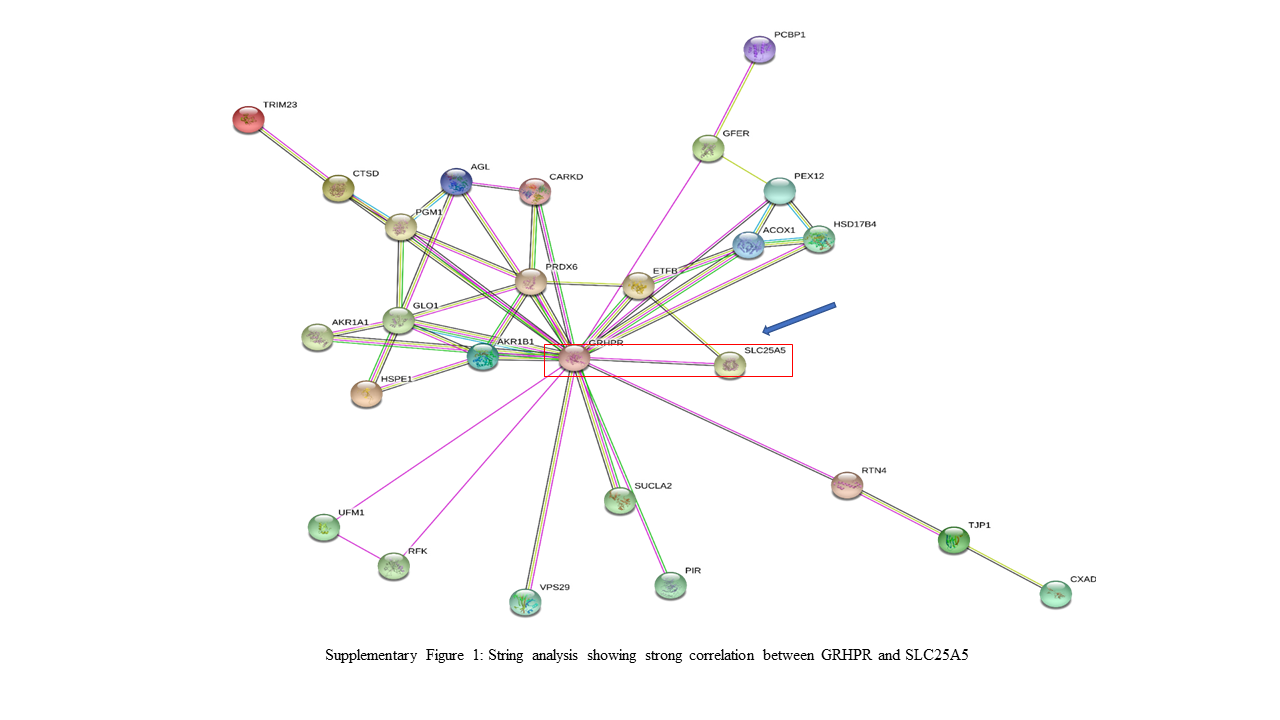


Supplementary Figure 4: String analysis showing Strong correlation between GRHPR and SLC25A5

References

Cloutier, J., Villa, L., Traxer, O., & Daudon, M. (2015). Kidney stone analysis: “Give me your stone, I will tell you who you are!” *World Journal of Urology*, *33*(2), 157–169. https://doi.org/10.1007/s00345-014-1444-9

Daudon, M., Jungers, P., & Bazin, D. (2008). Peculiar Morphology of Stones in Primary Hyperoxaluria. *Https://Doi.Org/10.1056/NEJMc0800990*, *359*(1), 100–102. https://doi.org/10.1056/NEJMC0800990

Racek, M., Racek, J., & Hupáková, I. (2019). Scanning electron microscopy in analysis of urinary stones. *Scandinavian Journal of Clinical and Laboratory Investigation*, *79*(3), 208–217. https://doi.org/10.1080/00365513.2019.1578995

Singh, V. K., & Rai, P. K. (n.d.). *Kidney stone analysis techniques and the role of major and trace elements on their pathogenesis: a review*. https://doi.org/10.1007/s12551-014-0144-4

| Sample ID | Age in Years | Gender | Onset of Disease | Familial history (M=mother, F=father, S=siblings)  Present =1; Absent=0; unknown=? | Consanguinity | Urban/Rural  residence | Self-history of kidney stone | Clinical phenotype  Other than KSD |
| --- | --- | --- | --- | --- | --- | --- | --- | --- |
| KS1 | 13 | Male | 8 | M0, F0 | No | Urban | Yes | Hyperoxaluria |
| KS2 | 10 | Female | 6 | M0, F? | No | Rural | No | No data available |
| KS3 | 2 | Male | 2 | M0, F? | No | Urban | No | Renal tubular dysfunction |
| KS4 | 12 | Female | 8 | M0, F0 | No | Rural | Yes | Hyperoxaluria |
| KS5 | 15 | Female | 11 | M1, F? | No | Urban | No | No Data available |
| KS6 | 17 | Female | 11 | M1, F? | No | Unknown | Yes | Hyperoxaluria |
| KS7 | 15 | Male | 13 | M1, F0 | No | Urban | Yes | Not found |
| S4 | 12 | Female | 10 | M0, F0 | No | Rural | Yes | Hyperoxaluria |
| S13 | 25 | Female | 14 | M1, F? | No | Rural | Yes | Hyperoxaluria |
| KS8 | 7 | Male | 6 | M? F? | No | Urban | No | No data available |
| KS9 | 2 | Male | 2 | 0 | No | Rural | No | No data available |
| KS10 | 2 | Male | 2 | M1, F? | No | Urban | No | Hyperoxaluria |
| KS11 | 3 | Female | 3 | ? | No | Rural | No | Not found |
| KS12 | 13 | Male | 9 | M0, F0 | No | Urban | Yes | CKD, Hyperoxaluria |
| KS13 | 12 | Male | 11 | F1 | No | Rural | Yes | Not found |
| KS14 | 6 | Female | 6 | M0, F0 | No | Rural | No | Hyperoxaluria, Hydronephrosis |
| KS15 | 10 | Male | 9 | M0,F0 | No | Urban | No | Hydronephrosis |
| KS16 | 10 | Male | 10 | M0, F0 | No | Rural | Yes | Not found. |

**Supplementary Table 1-** **Details of lower age group (pediatric)patient sample.**

| **Gene** | **Causative disease** | **Age of onset** | **Source** |
| --- | --- | --- | --- |
| SERPINH1 | Severe osteogenesis imperfecta | Infancy, neonatal | OphraNet |
| FBN1 | Progeroid and marfanoid aspect-lipodystrophy syndrome | Infancy, neonatal, Childhood | OphraNet |
| HSPG2 | Burtons syndrome/ Osteochondromuscular dystrophy | Antenatal/ neonatal/ Childhood | OphraNet |
| CRTAP | Lethal osteogenesis imperfecta | Infancy/ neonatal | OphraNet |
| CACNA1D | Sinoatrial node dysfunction and deafness | Infancy | OphraNet |
| USP8 | Pituitary adenoma 4 | Infancy | OMIM |
| COL1A2 | Severe osteogenesis imperfecta | Infancy/ neonatal | OphraNet |
| VIPAS9 | ARC, syndrome. Renal tubular dysfunction and neonatal cholestasis | Neonatal | OphraNet |
| ATP6V1B2 | Zimmermann-Laband syndrome 2 | Infancy, neonatal, Childhood | OphraNet |
| VDR | Rickets, vitamin D-resistant, type IIA | Infancy, neonatal | OphraNet |
| SLC34A1 | Cystinuria | All age groups | OphraNet |
| ACVRL1 | Rendu-Osler disease | All age groups | OphraNet |
| ATP6V1B1 | Renal tubular acidosis | Childhood | OphraNet |
| ATP7B | Hepatolenticular degeneration | Adolescent, adult, elderly, Childhood | OphraNet |
| CLIP2 | Williams-Beuren syndrome | --- | OMIM |

**Supplementary Table 2: All additional genes discovered in the study cohort with Nephrolithiasis, along with the disease and age of onset.**
